# Complete genome sequence of *Ferrimonas balearica* type strain (PAT^T^)

**DOI:** 10.4056/sigs.1161239

**Published:** 2010-10-27

**Authors:** Matt Nolan, Johannes Sikorski, Karen Davenport, Susan Lucas, Tijana Glavina Del Rio, Hope Tice, Jan-Fang Cheng, Lynne Goodwin, Sam Pitluck, Konstantinos Liolios, Natalia Ivanova, Konstantinos Mavromatis, Galina Ovchinnikova, Amrita Pati, Amy Chen, Krishna Palaniappan, Miriam Land, Loren Hauser, Yun-Juan Chang, Cynthia D. Jeffries, Roxanne Tapia, Thomas Brettin, John C. Detter, Cliff Han, Montri Yasawong, Manfred Rohde, Brian J Tindall, Markus Göker, Tanja Woyke, James Bristow, Jonathan A. Eisen, Victor Markowitz, Philip Hugenholtz, Nikos C. Kyrpides, Hans-Peter Klenk, Alla Lapidus

**Affiliations:** 1 DOE Joint Genome Institute, Walnut Creek, California, USA; 2 DSMZ - German Collection of Microorganisms and Cell Cultures GmbH, Braunschweig, Germany; 3 Los Alamos National Laboratory, Bioscience Division, Los Alamos, New Mexico, USA; 4 Biological Data Management and Technology Center, Lawrence Berkeley National Laboratory, Berkeley, California, USA; 5 Oak Ridge National Laboratory, Oak Ridge, Tennessee, USA; 6 HZI – Helmholtz Centre for Infection Research, Braunschweig, Germany; 7 University of California Davis Genome Center, Davis, California, USA

**Keywords:** chemoorganotroph, iron(III)-reducing bacterium, facultatively anaerobic, *Ferrimonadaceae*, *Gammaproteobacteria*, GEBA

## Abstract

*Ferrimonas balearica* Rossello-Mora *et al*. 1996 is the type species of the genus *Ferrimonas*, which belongs to the family *Ferrimonadaceae* within the *Gammaproteobacteria*. The species is a Gram-negative, motile, facultatively anaerobic, non spore-forming bacterium, which is of special interest because it is a chemoorganotroph and has a strictly respiratory metabolism with oxygen, nitrate, Fe(III)-oxyhydroxide, Fe(III)-citrate, MnO_2_, selenate, selenite and thiosulfate as electron acceptors. This is the first completed genome sequence of a member of the genus *Ferrimonas* and also the first sequence from a member of the family *Ferrimonadaceae*. The 4,279,159 bp long genome with its 3,803 protein-coding and 144 RNA genes is a part of the *** G****enomic* *** E****ncyclopedia of* *** B****acteria and* *** A****rchaea * project.

## Introduction

Strain PAT^T^ (= DSM 9799 = CCM 4581) is the type strain of the species *Ferrimonas balearica*, which is the type species of its genus *Ferrimonas* [1,2]. Currently, there are five species in the genus *Ferrimonas* [3]. The generic name derives from the Latin word ‘*ferrum*’ meaning ‘iron’ and the Greek word ‘*monas*’ meaning ‘unit’, referring to an iron(III)-reducing cell. The species epithet is also derived from the Latin word ‘*balearica*’ meaning ‘of the Balearic Islands’, referring to the place where the strain was isolated [1]. *Ferrimonas* is the type genus of the family *Ferrimonadaceae* and one of two genera in the family *Ferrimonadaceae* [4]. Strain PAT^T^ was described in 1995 by Rossello-Mora *et al*. [1] who isolated the strain from the upper few centimeters of marine sediment of the Palma de Mallorca harbor, Spain [1,5]. Here we present a summary classification and a set of features for *F. balearica* PAT^T^, together with the description of the complete genomic sequencing and annotation.

## Classification and features

The 16S rRNA gene sequence of PAT^T^ is 99% identical to four culturable strains, which are reported in GenBank [6]. Two strains, A2A-18 (AB193752) and A3B-47-3 (AB193753), were isolated from marine sand [7]. The culturable strain S8-05 (EU620413) was isolated from Palk Bay sediment in Thondi, India and another strain with accession number AY158002 was isolated from Ala Wai Canal sediment in Honolulu, USA. The 16S rRNA gene of strain PAT^T^ shares 93.5-97.4% sequence identity with the sequences of the type strains from the other members of the family *Ferrimonadaceae* [8]. The environmental samples database (env_nt) contains the marine metagenome clone 1096626783183 (96% sequence identity, AACY020355234). The genomic survey sequences database (gss) contains the uncultured bacterium clone BYUP987.b1 (92%, EF996742), isolated from a fecal sample of adult woman who gave birth after 11 months [9]. Altogether, strains belonging to the species *F. balearica* or the genus *Ferrimonas* are rather rare in the habitats screened so far (status September 2010).

**Figure 1:**
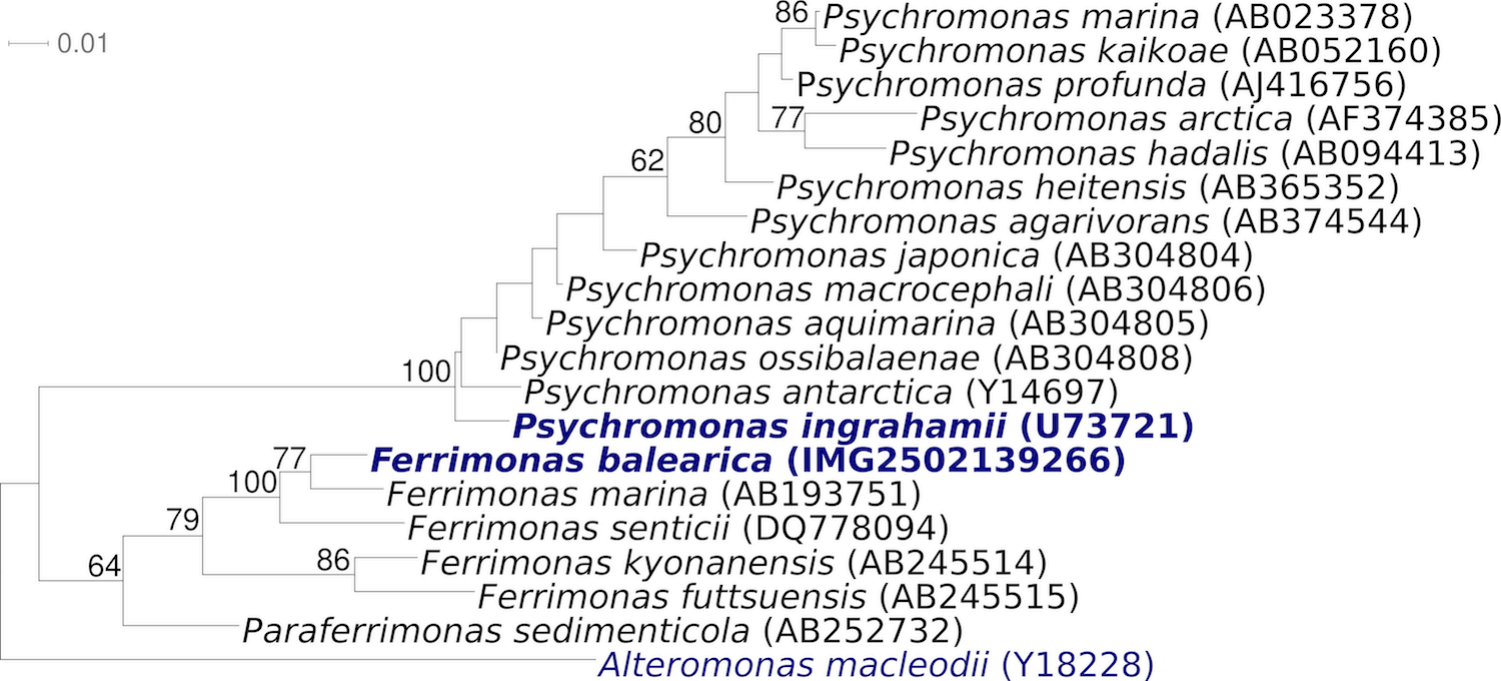
Phylogenetic tree highlighting the position of *F.* *balearica* PAT^T^ relative to the type strains of the other species within the family *Ferrimonadaceae* and to the type of the neighboring family *Psychromonadaceae*. The trees were inferred from 1,449 aligned characters [10,11] of the 16S rRNA gene sequence under the maximum likelihood criterion [12] and rooted with the type strain of the order *Alteromonadaceae*. The branches are scaled in terms of the expected number of substitutions per site. Numbers above branches are support values from 650 bootstrap replicates [13] if larger than 60%. Lineages with type strain genome sequencing projects registered in GOLD [14] are shown in blue, published genomes in bold (CP000510) [15].

Figure 1 shows the phylogenetic neighborhood of *F. balearica* PAT^T^ in a 16S rRNA based tree. The sequences of the seven 16S rRNA gene copies in the genome differ from each other by up to five nucleotides, and differ by up to four nucleotides from the previously published sequence (X93021), which contains two ambiguous base calls.

**Figure 2:**
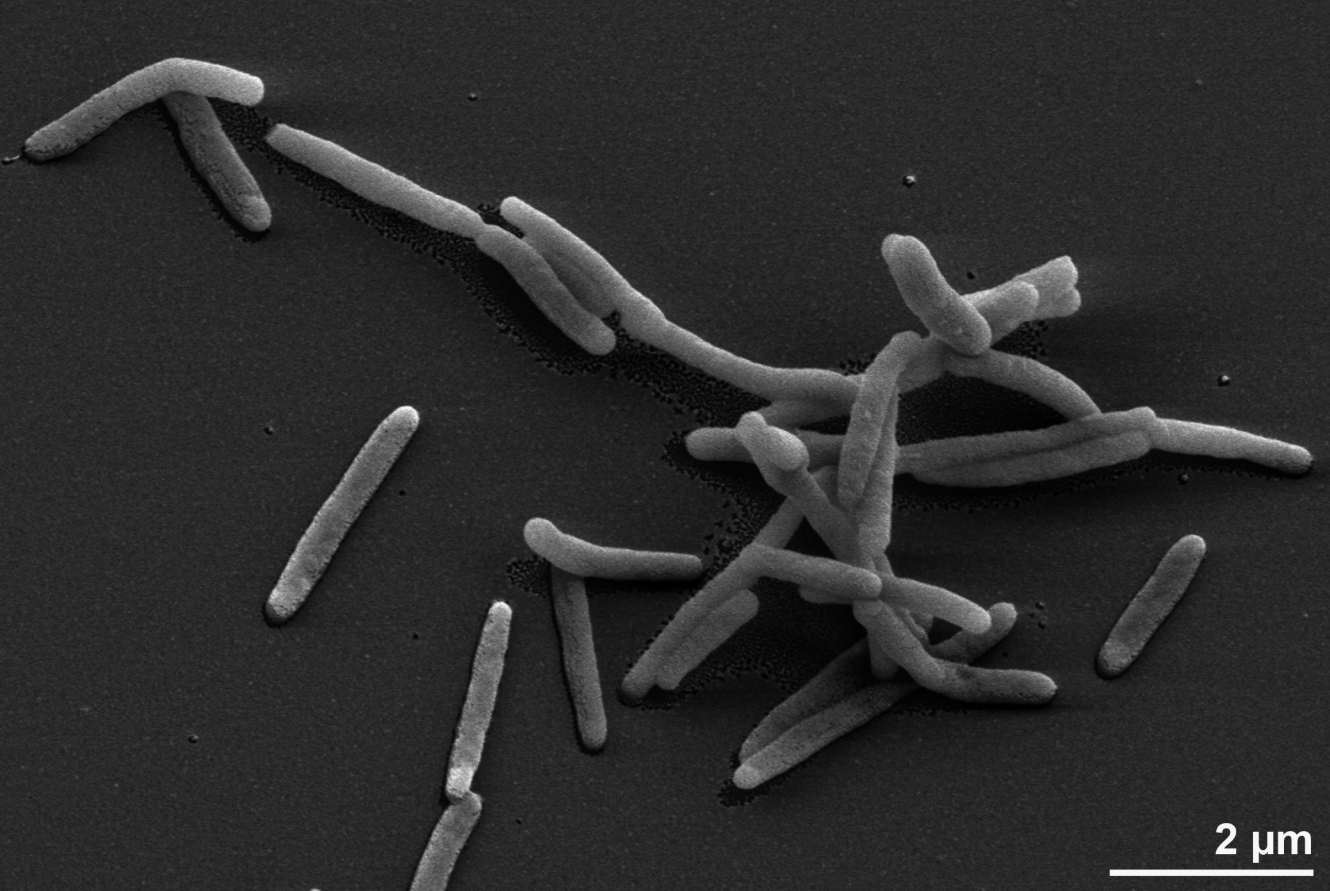
Scanning electron micrograph of *F. balearica* PAT^T^

Strain PAT^T^ is a Gram-negative, nonspore-forming, facultatively anaerobic bacterium [1]. The cells are straight rods (0.3-0.5 × 1.2-1.5 µm) with rounded ends (Figure 2, Table 1) [1,5] and appear singly, occasionally in pairs or short chains and usually not encapsulated [1,5]. Strain PAT^T^ is motile by means of monotrichous flagella (not visible in Figure 2, but 10% of the cells in the original liquid culture were highly motile) [1]. Colonies produce a black iron precipitate when the cells are grown on TSI agar [1]. Although initially isolated using TSI based media this strain grows better on Marine Broth. Colonies are often brown and mucous when the cells are grown under aerobic conditions [5]. Fresh isolates of this species may not form colonies on PYG agar medium, but the colonies are formed after several subcultivations in enrichment medium [1,5]. Resting stages of strain PAT^T^ are not known [5]. Cells of the strain undergo autolysis within five days under aerobic conditions [1,5]. Strain PAT^T^ does not contain polyhydroxybutyrate (PHB) or other intracellular inclusions [2]. The strain is chemoorganotrophic. Under anaerobic conditions, the reduction of Fe(III)-oxyhydroxide is coupled to the utilization of lactate as the electron donor, which yields magnetite [1,5]. Strain PAT^T^ uses oxygen, nitrate, Fe(III)-oxyhydroxide, Fe(III)-citrate, MnO_2_, selenate, selenite and thiosulfate as electron acceptors [1,5,25]. Strain PAT^T^ requires a minimum of 0.5% NaCl for growth, with a range of NaCl tolerance of 0.5%-7.5% [1]. It does not grow at 5°C or 44°C but does grow at 42°C [1]. The pH range for growth is 6-9 [1]. Enzymatic reactions are positive for catalase, oxidase, phenylalanine deaminase, DNAse and lipase (Tween 20 and Tween 80), but negative for amylase, arginine dihydrolase, gelatinase, lysine decarboxylase, Simmons citrate and urease [1,5]. The strain does not hydrolyze starch [1]. The genus *Ferrimonas* can be distinguished from other strictly respiratory Gram-negative genera of the *Gammaproteobacteria* based on its ability to reduce Fe(III), denitrification, growth at 42°C, presence of phenylalanine deaminase activity, inability to grow in NaCL-free media, lack of gelatinase, urease and a negative reaction of Simmons citrate test [5].

**Table 1:** Classification and general features of *F. balearica* PAT^T^ according to the MIGS recommendations [16].

**MIGS ID**	**Property**	**Term**	**Evidence code**
	Current classification	Domain *Bacteria*	TAS [17]
		Phylum *Proteobacteria*	TAS [18-20]
		Class *Gammaproteobacteria*	TAS [18,21]
		Order *Alteromonadales*	TAS [18,22]
		Family *Ferrimonadaceae*	TAS [4]
		Genus *Ferrimonas*	TAS [1,2]
		Species *Ferrimonas balearica*	TAS [1,2]
		Type strain PAT	TAS [1,2]
	Gram stain	negative	TAS [1]
	Cell shape	straight rods with rounded ends	TAS [1,5]
	Motility	yes	TAS [1]
	Sporulation	nonspore-forming	TAS [1]
	Temperature range	37°C-42°C	TAS [1,5]
	Optimum temperature	37°C	NAS
	Salinity	0.5%-7.5% (w/v) NaCl	TAS [1,5]
MIGS-22	Oxygen requirement	facultatively anaerobic	TAS [1]
	Carbon source	lactate	TAS [1]
	Energy source	chemoorganotroph	TAS [1,5]
MIGS-6	Habitat	marine sediment	TAS [1]
MIGS-15	Biotic relationship	free-living	NAS
MIGS-14	Pathogenicity	none	NAS
	Biosafety level	1	TAS [23]
	Isolation	marine sediment	TAS [1]
MIGS-4	Geographic location	Palma de Mallorca harbor, Spain	TAS [1]
MIGS-5	Sample collection time	1995 or before	TAS [1]
MIGS-4.1	Latitude	39.57	NAS
MIGS-4.2	Longitude	2.63	NAS
MIGS-4.3	Depth	not report	
MIGS-4.4	Altitude	below the sea level	TAS [1,5]

Evidence codes - IDA: Inferred from Direct Assay (first time in publication); TAS: Traceable Author Statement (i.e., a direct report exists in the literature); NAS: Non-traceable Author Statement (i.e., not directly observed for the living, isolated sample, but based on a generally accepted property for the species, or anecdotal evidence). These evidence codes are from of the Gene Ontology project [24]. If the evidence code is IDA, then the property was directly observed by one of the authors or an expert mentioned in the acknowledgements.

### Chemotaxonomy

The quinone profiles of strain PAT^T^ are MK-7 (62.9%), Q-8 (20.4%) and Q-7 (16%) [7]. The presence of both menaquinones and ubiquinones being indicative of the ability of this organism to grow aerobically (with ubiquinones) and anaerobically (with menaquinones). The presence of menaquinones and ubiquinones with different distributions of isoprenoid side chains is a feature also shared by members of the genus *Shewanella* [26-28] and *Paraferrimonas* [29] The major cellular fatty acids of strain PAT^T^, when grown on PYG medium, given in the original species description are C_17:1ω8_*_c_* (27.5%), iso-C_15:0_ (14.5%), C_17:0_ (7.8%), iso-C_13:0_ (5.8%), C_16:1ω7_*_c_* (4.7%), C_15:0_ (4.5%), C_13:0_ (4.5%), C_14:0_ (4.2%), C_18:1ω9_*_c_* (4.0%) and C_12:0_ 3-OH (1.8%), C_17:1ω6c_ (1.6%) and C_18:1ω7c_ (1.2%) [1]. More recent data show a somewhat different fatty acid pattern [7], with the fatty acids comprising iso-C_15:0_ (9.8%), C_15:0_ (1.8%) iso-C_16:1ω9c_ (10.4%) iso-C_16: ω7c_ (5.2%), C_16:0_ (13.4%) iso-C_17:0_ (2.1%) C_17:1ω8c_ (12.6%) C_17:0_ (7.9%) C_18:1ω9c_ (17.6%) C_18:1ω7c_ (4.9%) and C_18:0_ (3.9%). Hydroxylated fatty acids were not reported. Interestingly the fatty acids reported in a subsequent paper [25] that are based on the work of Kasuta *et al.* [7] omit the iso-C_16:1_ fatty acids. The fatty acids reported in the original publication [1] show a number of features also found in members of the genera *Shewanella* and *Paraferrimonas* [29,30]. Data generated in the DSMZ during the course of this work indicates that the fatty acids comprise, iso-C_13:0_ (3.7%), C_13:0_ (2.7%), C_12:0_ 3OH (2.2%), iso-C_14:0_ (1.1%), C_14:0_ (1.0%), iso-C_13:0_ 3OH (3.7%), C_13:0_ 3OH (1.9%), iso-C_15:0_ (16.1%), C_15:1 w8c_ (2.1%), C_15:0_ (4.5%), C_14:0_ 3-OH (2.9%), C_16:1 w9c_ (8.1%), C_16:1w7c_ (4.9%), C_16:0_ (8.4%), iso-C_15:0_ 3OH, (0.9%), iso-C_17:0_ (1.4%), C_17:1_ _w8c_ (14.7%), C_17:0_ (5.6%), C_18:1 w9c_ (7.8%) and C_18:1 w7c_ (1.4%). These results are more consistent with those published in the original description [1], but there are differences that cannot be attributed to differences in the growth conditions. The complete absence of hydroxylated fatty acids in the work of Kasuta *et al.* [7] suggests that no attempt was made to detect them. The presence of at least two positional isomers in unsaturated fatty acids with the same chain length is indicative of the presence of at least two enzymatic pathways for introducing the double bonds. A fairly simple polar lipid pattern has been reported for *Ferrimonas futtsuensis,* comprising, phosphatidylglycerol, phosphatidylethanolamine and an unidentified aminophopsholipid [29].

## Genome sequencing and annotation

### Genome project history

This organism was selected for sequencing on the basis of its phylogenetic position [31], and is part of the *** G****enomic* *** E****ncyclopedia of* *** B****acteria and* *** A****rchaea * project [32]. The genome project is deposited in the Genome OnLine Database [14] and the complete genome sequence is deposited in GenBank. Sequencing, finishing and annotation were performed by the DOE Joint Genome Institute (JGI). A summary of the project information is shown in Table 2.

**Table 2:** Genome sequencing project information

**MIGS ID**	**Property**	**Term**
MIGS-31	Finishing quality	Finished
MIGS-28	Libraries used	Two genomic Sanger libraries: 8 kb pMCL200 library, fosmid (40 kb) library
MIGS-29	Sequencing platforms	ABI3730
MIGS-31.2	Sequencing coverage	9.8 × Sanger
MIGS-30	Assemblers	Phrap
MIGS-32	Gene calling method	Prodigal 1.4, GenePRIMP
	INSDC ID	CP002209
	Genbank Date of Release	October 1, 2010
	GOLD ID	Gc01378
	NCBI project ID	30799
	Database: IMG-GEBA	2502082106
MIGS-13	Source material identifier	DSM 9799
	Project relevance	Tree of Life, GEBA

### Growth conditions and DNA isolation

*F. balearica* PAT^T^, DSM 9799, was grown in DSMZ medium 514 (Bacto Marine Broth) [33] at 28°C. DNA was isolated from 0.5-1 g of cell paste using Qiagen Genomic 500 DNA Kit (Qiagen, Hilden, Germany) following the standard protocol as recommended by the manufacturer, with modification st/L for cell lysis as described in Wu *et al*. [32].

### Genome sequencing and assembly

The genome was sequenced using the Sanger sequencing platform (6 and 40 kb DNA libraries). All general aspects of library construction and sequencing performed at the JGI can be found at http://www.jgi.doe.gov/. The Phred/Phrap/Consed software package was used for sequence assembly and quality assessment (www.phrap.com). After the shotgun stage, reads were assembled with parallel phrap (High Performance Software, LLC). Possible mis-assemblies were corrected with Dupfinisher or transposon bombing of bridging clones (Epicentre Biotechnologies, Madison, WI) [34]. Gaps between contigs were closed by editing in Consed, custom primer walk or PCR amplification. A total of 404 additional custom primer reactions were necessary to close gaps and to raise the quality of the finished sequence. The completed genome sequence contains 48,554 reads, achieving an average of 9.8-fold sequence coverage with an error rate less than 1 in 100,000.

### Genome annotation

Genes were identified using Prodigal [35] as part of the Oak Ridge National Laboratory genome annotation pipeline, followed by a round of manual curation using the JGI GenePRIMP pipeline [36]. The predicted CDSs were translated and used to search the National Center for Biotechnology Information (NCBI) nonredundant database, UniProt, TIGRFam, Pfam, PRIAM, KEGG, COG, and InterPro databases. Additional gene prediction analysis and functional annotation was performed within the Integrated Microbial Genomes - Expert Review (IMG-ER) platform [37].

## Genome properties

**Figure 3:**
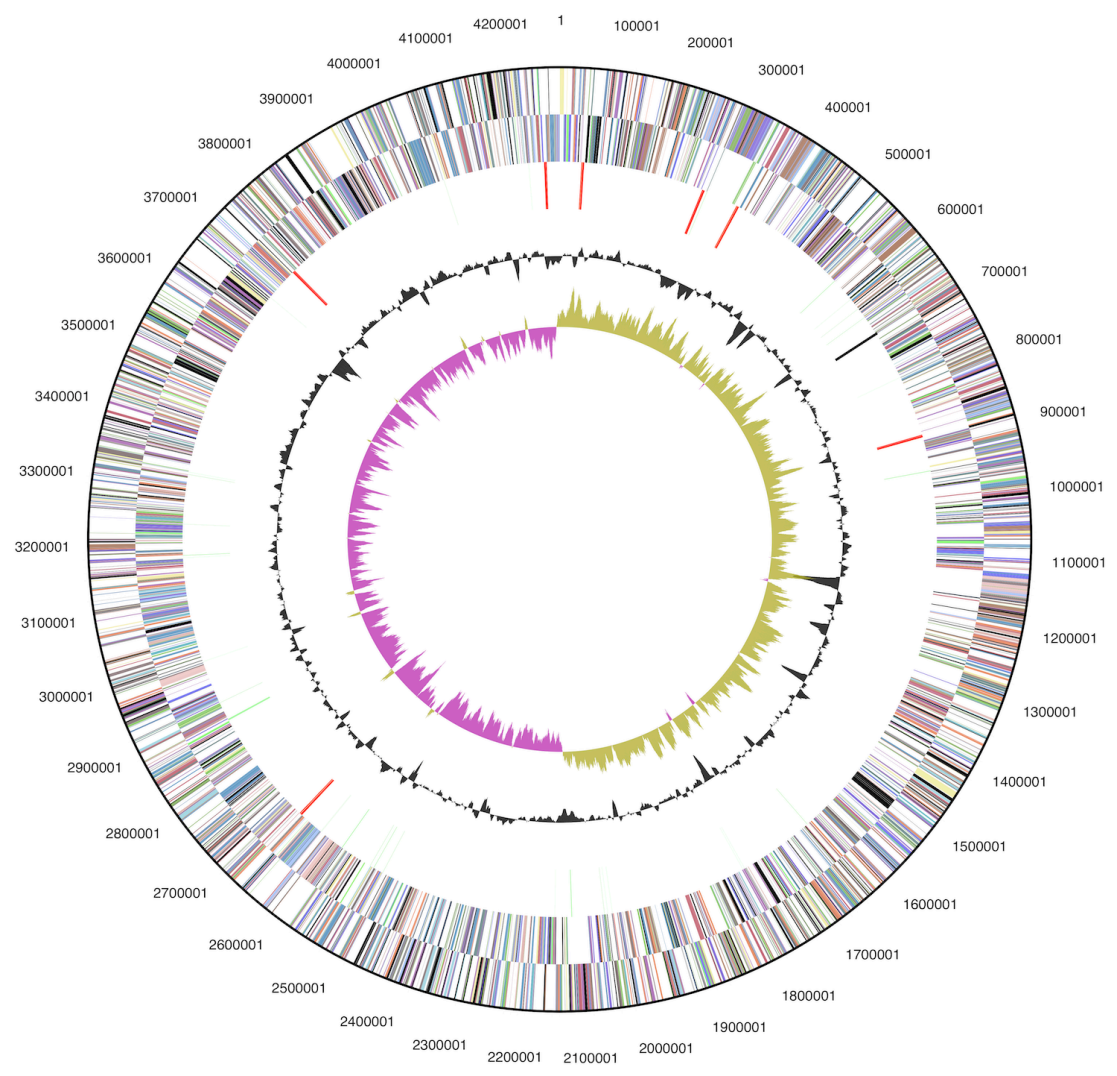
Graphical circular map of the genome. From outside to the center: Genes on forward strand (color by COG categories), Genes on reverse strand (color by COG categories), RNA genes (tRNAs green, rRNAs red, other RNAs black), GC content, GC skew.

The genome consists of a 4,279,159 bp long chromosome with a 60.2% GC content (Table 3 and Figure 3). Of the 3,947 genes predicted, 3,803 were protein-coding genes, and 144 RNAs; twenty one pseudogenes were also identified. The majority of the protein-coding genes (72.5%) were assigned a putative function while the remaining ones were annotated as hypothetical proteins. The distribution of genes into COGs functional categories is presented in Table 4.

**Table 3:** Genome Statistics

**Attribute**	**Value**	**% of Total**
Genome size (bp)	4,279,159	100.00%
DNA coding region (bp)	3,842,563	89.80%
DNA G+C content (bp)	2,576,887	60.22%
Number of replicons	1	
Extrachromosomal elements	0	
Total genes	3,947	100.00%
RNA genes	144	3.65%
rRNA operons	7	
Protein-coding genes	3,803	96.35%
Pseudo genes	21	0.53%
Genes with function prediction	2,860	72.46%
Genes in paralog clusters	462	11.71%
Genes assigned to COGs	2,929	74.21%
Genes assigned Pfam domains	3,089	78.26%
Genes with signal peptides	1,154	29.24%
Genes with transmembrane helices	981	24.85%
CRISPR repeats	0	

**Table 4:** Number of genes associated with the general COG functional categories

**Code**	**value**	**%age**	**Description**
J	189	5.8	Translation, ribosomal structure and biogenesis
A	1	0.0	RNA processing and modification
K	213	6.5	Transcription
L	138	4.2	Replication, recombination and repair
B	1	0.0	Chromatin structure and dynamics
D	35	1.1	Cell cycle control, cell division, chromosome partitioning
Y	0	0.0	Nuclear structure
V	61	1.9	Defense mechanisms
T	178	5.5	Signal transduction mechanisms
M	219	6.7	Cell wall/membrane/envelope biogenesis
N	133	4.1	Cell motility
Z	0	0.0	Cytoskeleton
W	0	0.0	Extracellular structures
U	128	3.9	Intracellular trafficking and secretion, and vesicular transport
O	155	4.8	Posttranslational modification, protein turnover, chaperones
C	238	7.3	Energy production and conversion
G	105	3.2	Carbohydrate transport and metabolism
E	248	7.6	Amino acid transport and metabolism
F	85	2.6	Nucleotide transport and metabolism
H	167	5.1	Coenzyme transport and metabolism
I	99	3.0	Lipid transport and metabolism
P	184	6.7	Inorganic ion transport and metabolism
Q	53	1.6	Secondary metabolites biosynthesis, transport and catabolism
R	338	10.4	General function prediction only
S	287	8.8	Function unknown
-	1,018	25.8	Not in COGs
